# Investigating the resistome of haemolytic bacteria in Arctic soils

**DOI:** 10.1111/1758-2229.70028

**Published:** 2024-10-23

**Authors:** Diana C. Mogrovejo‐Arias, Melanie C. Hay, Arwyn Edwards, Andrew C. Mitchell, Jörg Steinmann, Florian H. H. Brill, Bernd Neumann

**Affiliations:** ^1^ MicroArctic Research, Dr. Brill + Partner GmbH Institut für Hygiene und Mikrobiologie Hamburg Germany; ^2^ Institute of Biological, Environmental & Rural Sciences (IBERS) Aberystwyth University Aberystwyth UK; ^3^ Department of Pathobiology and Population Sciences The Royal Veterinary College Brookmans Park UK; ^4^ Department of Geography and Earth Sciences Aberystwyth University Aberystwyth UK; ^5^ Institute of Clinical Microbiology, Infectious Diseases and Infection Control Paracelsus Medical University, Klinikum Nürnberg Nuremberg Germany

## Abstract

Microorganisms inhabiting hostile Arctic environments express a variety of functional phenotypes, some of clinical interest, such as haemolytic ability and antimicrobial resistance. We studied haemolytic bacterial isolates from Arctic habitats, assessing their minimum inhibitory concentration (MIC) against antimicrobials. We then performed whole genome sequencing and analysed them for features conferring antimicrobial resistance. MIC data showed that *Micromonospora* spp. belong to 33% non‐wild type (NWT) for erythromycin and penicillin and 22% NWT for tetracycline. Both *Pseudomonas* spp. belong to 43% NWT for nalidixic acid and streptomycin and 29% NWT for colistin. Finally, the *Pedobacter* isolate was in 80% NWT for antimicrobials tested. Whole‐genome sequencing analyses revealed that fluoroquinolones, tetracyclines, macrolides and penams were the most frequent drug classes against which genotypic resistance was found. Additionally, resistance genes to heavy metals and disinfectants were identified. Our research demonstrates the presence of antimicrobial resistance in bacteria from Arctic habitats and highlights the importance of conservation efforts in these environments, where anthropogenic influence is becoming more evident. Furthermore, our data suggest the possible presence of novel resistance mechanisms, which could pose a threat if the responsible genes are transferable between species or become widespread due to environmental stress and alterations brought about by climate change.

## Graphical abstract

The study explored the antimicrobial potential of bacteria isolated from Arctic soil. The findings revealed that these bacteria harbour a multitude of resistance genes against antibiotics and disinfectants. Additionally, the bacteria demonstrated the ability to cause haemolysis and grow at body temperature. With the progression of climate change, bacteria from Arctic environments could pose a significant threat to human health.

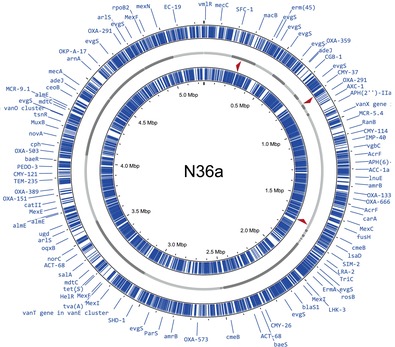

## INTRODUCTION

Cold‐adapted microorganisms inhabit a large proportion of Earth's ecosystem (Sogin et al., 2006; Tindall, 2004). For instance, most oceans have an average higher temperature of 5°C and the Polar Regions alone represent 20% of the world's land area (Casanueva et al., 2010; Malard & Pearce, 2018). This study focused on Arctic bacteria, following the open question how likely these species pose a threat to human health. An essential precondition to this topic is the progressing climate change that leads bacteria of former permafrost to adapt to constantly increased temperatures. Other than expected, Arctic bacteria seem not to be extremophile and might easily adjust to new ecological conditions (Maccario et al., 2015). It is known that environmental bacteria can gain clinically relevant resistance traits, for example, through migrating birds and afterwards become human pathogens, for example, *Aeromonas* sp. (Lamy et al., 2022; Segawa et al., 2013). Recent research continually elucidates the growing potential of environmental bacteria and their possible new role in human health, especially their virulence and resistance assets and acquisitions (Depta & Niedźwiedzka‐Rystwej, 2023; Perron et al., 2015).

Naturally bacterial communities evolve genes aimed at providing fitness advantages to the producing organisms either by reducing competition, favouring the acquisition of nutrients or aiding with inter‐microbial communication (Falkow, 2004; Martínez, 2008). This probably led to virulence factors, for example, bacteriocin and antimicrobial resistance genes, since antimicrobial compounds, also clinically used antibiotics, were produced by environmental microbes (Martinez, 2009). Even though genes that confer resistance to antimicrobials are widespread in natural environments, it could be shown that these genes, as well as resistant bacterial species, were introduced by anthropogenic factors into polar environments (Miller et al., 2009; Segawa et al., 2013). Despite resistance, phenotypes considered as potentially pathogenic, for example, the production of haemolytic enzymes, are studied in Arctic isolates and provide broad insights into the functional diversity of polar microorganisms (Mogrovejo et al., 2020; Mogrovejo‐Arias et al., 2020; Perini et al., 2019). Beta‐haemolytic enzymes are widespread throughout the entire bacterial phylogeny and well known for their impact virulence factors in pathogenesis (Mogrovejo et al., 2020; Thelestam & Ljungh, 1981).

For this study, we selected five beta‐haemolytic bacterial strains, namely two *Micromonospora* spp., two *Pseudomonas* spp. and one *Pedobacter* sp., isolated from Arctic environments. These isolates were part of our previous study and selected for further investigations based on their unsuspected abilities to thrive at 37°C and their virulence potential of beta‐haemolysis (Mogrovejo et al., 2020). Gram‐positive species of the genus *Micromonospora*, referred to as “rare actinomycetes,” are common producers of secondary metabolites showing novel antimicrobial activities (Qi et al., 2020). Species from Gram‐negative genera, such as *Pedobacter*, are of particular interest as they have been recently described as environmental “superbugs” due to their resistance against beta‐lactams, aminoglycosides and polymyxins (Viana et al., 2018). Other Gram‐negative species as of the genus *Pseudomonas*, especially *P. aeruginosa*, have been extensively studied for their considerable antibiotic resistance profiles while other species remain less characterized albeit still clinically relevant (Aeschlimann, 2003; Battle et al., 2009).

This study aimed to improve the understanding of Arctic bacteria showing the potential of becoming One Health pathogens. The primary focus was laid on in vitro antibiotic resistance measurements combined with in silico genetic predictions. Thus, a minimal inhibitory concentration (MIC) testing of antibiotics classified by the World Health Organization as critically important for human medicine should be appointed for environmental bacteria lacking breakpoint rules. In addition, genome sequencing was performed to identify genetic determinants that are likely responsible for and general resistance capabilities, including heavy metals and disinfectants, since cold‐adapted environmental bacteria have been identified as important reservoirs for various resistance genes (Sajjad et al., 2023; Vats et al., 2022). These two features will be included since new understandings of resistance mechanisms and resistance evolution becoming increasingly essential (Wang et al., 2024).

## EXPERIMENTAL PROCEDURES

### Bacterial isolates

Five bacterial isolates of our previous study were used in this study (see Table 1) (Mogrovejo‐Arias et al., 2020). These isolates were selected since they consistently exhibited haemolytic activity, which is generally regarded as a potentially pathogenic phenotype, as summed up in Table 1.

**TABLE 1:** Characterization of bacterial isolates used in this study, which have been isolated from Mogrovejo‐Airas et al. (Mogrovejo et al., 2020; Mogrovejo‐Arias et al., 2020).

Isolate (GenBank accession number)	Closest GenBank taxonomic 16S rRNA neighbour (GenBank accession number)	% identity closest 16S rRNA neighbour	Isolated from	Isolation temperature	Haemolysis on bovine blood agar		Haemolysis on sheep blood agar	
					15°C	30°C	15°C	30°C
S3 (MH714651)	*Micromonospora aurantiaca* (NR_074415.1)	99%	Marine sediment	37°C	γ	β	γ	β
N18 (MH714618)	*Micromonospora chalcea* (NR_118842.1)	99%	Soil	37°C	β	β+	β	β
N36a (MH714626)	*Pedobacter nyackensis* (NR_044380.1)	99%	Sediment	17°C	α	α	β+	α
N40 (MH714635)	*Pseudomonas helmanticensis* (NR_126220.1)	98%	Soil	5°C	β+	NG	β	NG
N71 (MH714637)	*Pseudomonas lurida* (NR_042199.1)	99%	Soil	5°C	β+	NG	β+	NG

Abbreviations: α, α‐haemolytic; β, β‐haemolytic; γ, non‐haemolytic; +, a stronger phenotype; −, a weaker phenotype; NG, no growth.

### Determination of minimum inhibitory concentration

The following 11 medically relevant antimicrobials, from seven different drug classes were used: ampicillin, ciprofloxacin, colistin, erythromycin, gentamicin, kanamycin, penicillin, streptomycin, tetracycline and vancomycin. All were purchased from Carl Roth®, Germany, except ciprofloxacin (Sigma‐Aldrich®, Germany). The range for MIC determination (mg L^−1^) of the antimicrobials was 0.0125–256 mg L^−1^, except for ciprofloxacin (0.00625–32 mg L^−1^) and streptomycin (0.0625–1024 mg L^−1^). The experiments were set up following the standardized methodologies described in using *Bacillus subtilis* subsp. spizizenii (ATCC® 6633™) and *Pseudomonas aeruginosa* (ATCC® 15442™) as control strains (Wiegand et al., 2008). Colistin and nalidixic acid were not tested for the *Micromonospora* spp. (S3, N18) as well as vancomycin for the *Pedobacter* sp. (N36a), and ampicillin, erythromycin, penicillin and vancomycin for the *Pseudomonas* spp. (N40, N71) since these are intrinsically resistant to respective substances, according to EUCAST expert rules (http://www.eucast.org/expert_rules_and_intrinsic_resistance/).

For the Gram‐positive isolates, the agar dilution method was used with Mueller Hinton broth (Carl Roth®) supplemented with 17 g of agar (pH 7.2–7.4, 25°C) and stock solutions of the antimicrobials at 10 mg L^−1^. Agar plates were not supplemented with Ca^2+^ or Mg^2+^ and they were prepared and used on the same day and incubated at 37°C for 20 h after inoculation. Single colonies from an overnight culture on nutrient agar were suspended in sterile saline solution and the turbidity adjusted to a 0.5 McFarland standard using a Turbidimeter (Grant Instruments, UK). The suspension was plated on prepared solid agar, using sterile cotton swabs. Two agar plates without antibiotic were inoculated per each isolate as viability controls.

Gram‐negative isolates were test using a broth microdilution protocol, with Mueller Hinton broth (Carl Roth®, pH 7.2–7.4, 25°C). The medium was supplemented with 20 mg L^−1^ of Ca^2+^ and 10 mg L^−1^ of Mg^2+^ before testing tetracycline for the *Pedobacter* sp. N36a and for gentamicin, kanamycin and streptomycin for the *Pseudomonas* spp. N40 and N71 (D'amato et al., 1975; Wiegand et al., 2008). A 0.5 McFarland bacterial suspension was diluted 1:100, of which 50 μL were used to inoculate 96‐well microtiter plates prepared with 50 μL of the respective antimicrobial dilutions for a final bacterial inoculum of 5 × 10^5^ CFU mL^−1^. As growth control served 60 μL of bacterial suspension. Viable colony counts were performed by withdrawing 10 μL of the growth well immediately after inoculation and diluting in 10 mL of sterile saline solution. After mixing, 100 μL were plated on nutrient agar and incubated at 37°C for 20 h. This step was standardly done to ensure the correct inoculum CFU.

### Interpretation of MIC values

No specific EUCAST breakpoints (Version 9.1 and 10.0) have been defined for the organism‐antimicrobial combinations studied here and thus, the terms “Resistant,” “Susceptible” and “Intermediate” were not used (Schwarz et al., 2010). Instead, ECOFFs were used to describe the observed MIC distributions, using the EUCAST “Antimicrobial wild type distributions of microorganisms” database (available at: https://mic.eucast.org/Eucast2/). The ECOFF is described as the highest MIC value of organisms with no phenotypically detectable acquired resistance mechanisms and it is useful to define the upper end of the wild‐type MIC distribution. The isolates were considered as wild‐type (WT) when their MIC was lower or equal to the ECOFF established for each antibiotic. Alternatively, isolates were described as “belonging to the non‐WT distribution” (NWT) when the MIC for the corresponding antimicrobial was greater than the ECOFF. For the *P. aeruginosa* isolate, the MICs/ECOFFs for “*Pseudomonas aeruginosa*” were applied. Since the species specific ECOFF values for four isolates tested were not listed in the EUCAST database, the ECOFF values of related species (same genus/phylum) for each antimicrobial were used (https://mic.eucast.org/Eucast2/). For this, the values for several relevant genera were compared and to extrapolate a “consensus value” usable for the environmental species (Table 2). The decisions were made on a conservative approximation, so the highest value for a genus was used. In detail, each antimicrobial was searched in the EUCAST database and a list of bacterial genera with an ECOFF value was generated. From this list, we determined a consensus value that (a) encompassed as many relevant genera as possible, and/or that (b) was the highest value for the corresponding type of bacteria (see Table 2). For two antibiotics, two different ECOFFs were used due to the differences between Gram‐positive and Gram‐negative bacteria. Intrinsic resistance to a certain antibiotic were not tested (NT).

**TABLE 2:** Overview of used consensus ECOFF values and genera.

Antimicrobial	Consensus ECOFF value	For Gram positives	For Gram negatives	Genera taken in comparison for consensus values
AMP	8	4	8	*Campylobacter*, *Citrobacter*, *Enterococcus*, *Escherichia*, *Haemophilus*, *Klebsiella*, *Listeria*, *Moraxella*, *Pasteurella*, *Proteus*, *Salmonella*, *Shigella*, *Streptococcus*
CIP	4	4	0.125 (4 for N36)	*Acinetobacter*, *Campylobacter*, *Citrobacter*, *Enterobacter*, *Enterococcus*, *Escherichia*, *Haemophilus*, *Hafnia*, *Helicobacter*, *Klebsiella*, *Moraxella*, *Neisseria*, *Pasteurella*, *Proteus*, *Pseudomonas*, *Salmonella*, *Staphylococcus*, *Streptococcus*, *Yersinia*
CST	4	n/a	4	*Acinetobacter*, *Enterobacter*, *Escherichia*, *Klebsiella*, *Pseudomonas*
ERY	1	1	16	*Legionella*, *Listeria*, *Moraxella*, *Neisseria*, *Staphylococcus*, *Streptococcus*
GEN	4	32	4	*Acinetobacter*, *Campylobacter*, *Citrobacter*, *Enterobacter*, *Escherichia*, *Haemophilus*, *Klebsiella*, *Morganella*, *Proteus*, *Salmonella*, *Serratia*, *Staphylococcus*
KAN	8	8	8	*Escherichia*, *Staphylococcus*
NAL	16	n/a	16	*Acinetobacter*, *Campylobacter*, *Escherichia*, *Salmonella*
PEN	0.125 and 32	0.125	32	For 0.125 *Clostridium*, *Propionibacterium*, *Staphylococcus*, *Streptococcus* For 32 *Enterococcus* Note: PEN has no effect on Gram negs, so the highest ECOFF value available was used, which was 32 for Enterococcus
STR	16	16	16	*Campylobacter*, *Escherichia*, *Salmonella*, *Staphylococcus*
TET	4 and 8	4	8	For 4: *Clostridium*, *Enterococcus*, *Listeria*, *Staphylococcus*, *Streptococcus* For 8: *Acinetobacter*, *Bordetella*, *Campylobacter*, *Citrobacter*, *Escherichia*, *Haemophilus*, *Helicobacter*, *Klebsiella*, *Mannheimia*, *Moraxella*, *Morganella*, *Pasteurella*, *Salmonella*, *Yersinia* Note: There was great variability in the Gram neg and Gram pos values. Consensus values of 4 and 8 included the majority of medical important genera for each type
VAN	4	4	n/a	*Clostridium*, *Enterococcus*, *Propionibacterium*, *Staphylococcus*, *Streptococcus*

### Whole genome sequencing

Genomic DNA was extracted from overnight bacterial cultures on tryptic soy broth using the DNeasy Blood and Tissue extraction kit (QIAGEN Ltd., UK). The extracted DNA was quantified using a Qubit dsDNA broad range Assay Kit (ThermoFisher, Germany), purified using Ampure XP beads (AMPure XP beads, Beckman Coulter, UK) and sequenced using 2 × 250 bp paired‐end Illumina sequencing by MicrobesNG (MicrobesNG, UK). The MicrobesNG bioinformatics pipeline is summarized as follows: The closest available reference genome was identified using Kraken and reads were mapped to the reference genome using BWA mem to assess the quality of the data (Li & Durbin, 2009; Wood & Salzberg, 2014). Adapters were trimmed from raw reads using Trimmomatic 0.30 with a sliding window cut‐off of Q15 (Bolger et al., 2014). Reads were assembled de novo using SPAdes version 3.7 with default settings and mapped back to the contigs using BWA mem to get assembly quality metrics. Variant calling was performed using VarScan and automated annotation was performed using Prokka (Bankevich et al., 2012; Koboldt et al., 2009; Seemann, 2014).

### Analyses of the genomes

Whole‐genome relatedness and taxonomy were evaluated using the Kbase tool‐kit Fast_ANI on the Genome taxonomy database (Chaumeil et al., 2019; Jain et al., 2018). Additionally, the assembled genomes were submitted to the Comprehensive Genome Analysis Service of the Pathosystems Resource Integration Center (PATRIC) (https://www.patricbrc.org/) in order to annotate the genomes, while the amino acid fasta files were submitted to the RGI server of the Comprehensive Antibiotic Resistance Database (CARD) (https://card.mcmaster.ca/) to search for antimicrobial resistance genes (Alcock et al., 2020; Brettin et al., 2015; Wattam et al., 2017). To overcome limitations of databases, often solely including records from well‐known organisms, also the Bakta (v1.7.0) pipeline was used enabling alignment‐free genome annotations (Schwengers et al., 2021).

To determine relationships with the closest type strains, a two‐in‐one complementary approach based on 16S rRNA and whole genome sequences. The 16S rRNA gene sequence was extracted from the WGS‐based genome data by Type Strain Genome Server (TYGS) using RNAmmer and the sequence was compared with the 16S rRNA gene sequences of all type strains available in the TYGS database using BLAST (Camacho et al., 2009; Lagesen et al., 2007; Meier‐Kolthoff & Göker, 2019). Further, TYGS provided a whole‐genome‐based method for taxonomic classification by comparing the query genome with database of all type strain genomes, based on the technique of Genome‐to‐Genome Distance Calculator (Auch et al., 2006; Holland et al., 2002; Meier‐Kolthoff et al., 2013). Further, the BacDive database was used to compare genome parameters as GC content and genome size with type strains for plausibility (Reimer et al., 2022).

## RESULTS

### MIC determination and interpretation

The results of the MIC determination are summarized in Table 3. All viability controls contained between 24 and 71 CFU mL^−1^ and were within the recommendations of EUCAST.

**TABLE 3:** MIC testing results (in mg L^−1^) of five Arctic strains with beta‐haemolytic activity and assignment into wild type (WT) or non‐wild type (NWT).

Genus	Isolate	Antimicrobial										
		Beta‐lactam/penam		Quinolone		Polymyxin	Macrolide	Aminoglycoside			Tetracycline	Glycopeptide
		AMP	PEN	CIP	NAL	CST	ERY	GEN	KAN	STR	TET	VAN
*Micromonospora* spp.	S3	0.5 (WT)	2 (NWT)	0.25 (WT)	NT	NT	2 (NWT)	0.5 (WT)	2 (WT)	0.5 (WT)	8 (WT)	0.5 (WT)
	N18	2 (WT)	2 (NWT)	2 (WT)	NT	NT	2 (NWT)	4 (WT)	8 (WT)	2 (WT)	4 (WT)	2 (WT)
*Pedobacter* sp.	N36a	256 (NWT)	32 (WT)	8 (NWT)	>256 (NWT)	>256 (NWT)	>256 (NWT)	32 (NWT)	>256 (NWT)	256 (NWT)	4 (WT)	NT
*Pseudomonas* spp.	N40	NT	NT	0.12 (WT)	>256 (NWT)	<0.015 (WT)	NT	4 (WT)	2 (WT)	128 (NWT)	8 (WT)	NT
	N71	NT	NT	0.12 (WT)	>256 (NWT)	>256 (NWT)	NT	2 (WT)	2 (WT)	32 (NWT)	8 (WT)	NT

Abbreviations: AMP, ampicillin; CIP, ciprofloxacin; CST, colistin; ERY, erythromycin; GEN, gentamicin; NAL, nalidixic acid; NT, not tested (intrinsically resistant); PEN, penicillin G; STR, streptomycin; TET, tetracycline; VAN, vancomycin.

As shown in the table, the WT/NWT proportion varied for each species. For better comparing the results, the percentages of NWT were calculated by dividing the number of antibiotics that show an acquired resistance phenotype (NWT) against the antibiotics that were tested susceptible. The intrinsic resistances were excluded in this calculation. The *Micromonospora* spp. isolates (S3 and N18) showed NWT for 3/9 (33.33%) or 2/9 (22.22%) substances, respectively. Also the *Pseudomonas* spp. isolates (N40 and N71) showed NWT for 2/7 (28.75%) or 3/7 (42.85%) substances, respectively. The *Pedobacter* isolate (N36a) was in 80% NWT for tested antibiotics.

### Whole genome sequencing and annotation

Good‐quality genome sequences were obtained for all five isolates. Closest related species, sequencing metrics for the genomes and assemblies were provided by MicrobesNG (UK) and are presented in Table S1, Supporting Information while the results of the genome annotation with PATRIC are shown in Table S2. None of the isolates included plasmids. The genome sequences have been deposited in the National Center for Biotechnology Information (NCBI) database under the Bioproject number PRJNA659552. TYGS analyses revealed that *Pedobacter* N36a and *Pseudomonas* N40 have an average nucleotide identity of ≤95% compared to all genomes of this database and might constitute new species (Table S1). The species predictions for the other three strains remained plausible, based on TYGS analyses and comparing genome sizes and GC contents with the BacDive database. The genome annotation carried out with PATRIC identified up to 32 different antibiotic substance classes and combinations thereof against which the isolates had resistance genes.

### Antibiotic resistance prediction

CARD analysis was done selecting for perfect, strict and loose hits. Numerous matches were found for each of the isolates, ranging from a minimum of 398 hits for N36a to a maximum of 694 hits for N71. The majority of these, however, were loose matches. To capture more potential matches, a slightly relaxed cut‐off was established by dividing the best hit bit score of the query sequence by the curated BLASTP bit score of each Antibiotic Resistance Ontology (ARO) category and analysed only the resulting 46 matches whose scores were a percentage of 80% or higher (Table 4).

**TABLE 4:** High percentage score matches for antibiotic resistance determinants of five bacterial genomes found by CARD.

Isolate	Cut off	Percentage score	Pass bitscore	Best hit bitscore	Best hit ARO	Drug class^a^	Resistance mechanism	AMR gene family
S3	Loose	81.13	300	243.4	*poxtA*	macrolides; lincosamides; streptogramins; tetracyclines; oxazolidinones	antibiotic target protection	ABC‐F ATP‐binding cassette ribosomal protection protein
		81.43	420	342	*mtrA*	macrolides; penams	antibiotic efflux	resistance‐nodulation‐cell division (RND) antibiotic efflux pump
		81.65	200	163.3	*Pseudomonas aeruginosa* *soxR*	fluoroquinolones; cephalosporins; glycylcycline; penams; tetracyclines	antibiotic target alteration; antibiotic efflux	ATP‐binding cassette (ABC) antibiotic efflux pump; major facilitator superfamily (MFS) antibiotic efflux pump; resistance‐nodulation‐cell division (RND) antibiotic efflux pump
		87.27	700	610.9	*Escherichia coli* EF‐Tu mutants conferring resistance to Pulvomycin	elfamycins	antibiotic target alteration	elfamycin resistant EF‐Tu
		99.35	200	198.7	*Neisseria gonorrhoeae folP* with mutation conferring resistance to sulfonamides	sulfonamides	antibiotic target alteration	sulfonamide resistant dihydropteroate synthase folP
N18	Loose	81.17	420	340.9	*mtrA*	macrolides; penams	antibiotic efflux	resistance‐nodulation‐cell division (RND) antibiotic efflux pump
		81.9	300	245.7	*poxtA*	macrolides; lincosamides; streptogramins; tetracyclines; oxazolidinones	antibiotic target protection	ABC‐F ATP‐binding cassette ribosomal protection protein
		82.05	200	164.1	*Pseudomonas aeruginosa* *soxR*	fluoroquinolones; cephalosporins; glycylcycline; penams; tetracyclines	antibiotic target alteration; antibiotic efflux	ATP‐binding cassette (ABC) antibiotic efflux pump; major facilitator superfamily (MFS) antibiotic efflux pump; resistance‐nodulation‐cell division (RND) antibiotic efflux pump
		87.27	700	610.9	*Escherichia coli* EF‐Tu mutants conferring resistance to Pulvomycin	elfamycins	antibiotic target alteration	elfamycin resistant EF‐Tu
	Strict	101.5	200	203	*Neisseria gonorrhoeae* folP with mutation conferring resistance to sulfonamides	sulfonamides	antibiotic target alteration	sulfonamide resistant dihydropteroate synthase folP
N36a	Loose	83.31	700	583.2	*Escherichia coli EF‐Tu mutants conferring resistance to Pulvomycin*	elfamycins	antibiotic target alteration	elfamycin resistant EF‐Tu
		96.04	750	720.3	*adeF*	fluoroquinolones; tetracyclines	antibiotic efflux	resistance‐nodulation‐cell division (RND) antibiotic efflux pump
		97.53	750	731.5	*adeF*	fluoroquinolones; tetracyclines	antibiotic efflux	resistance‐nodulation‐cell division (RND) antibiotic efflux pump
		98.2	750	736.5	*adeF*	fluoroquinolones; tetracyclines	antibiotic efflux	resistance‐nodulation‐cell division (RND) antibiotic efflux pump
N40	Loose	80.08	1900	1521.5	*TriC*	triclosan	antibiotic efflux	resistance‐nodulation‐cell division (RND) antibiotic efflux pump
		80.65	170	137.1	*Pseudomonas aeruginosa emrE*	aminoglycosides	antibiotic efflux	small multidrug resistance (SMR) antibiotic efflux pump
		82.79	1950	1614.4	*MexB*	macrolides; fluoroquinolones; monobactam; carbapenem; cephalosporins	antibiotic efflux	resistance‐nodulation‐cell division (RND) antibiotic efflux pump
		83.59	800	668.7	*OprN*	fluoroquinolones; diaminopyrimidines; phenicols	antibiotic efflux	resistance‐nodulation‐cell division (RND) antibiotic efflux pump
		83.99	1900	1595.9	*MexW*	macrolides; fluoroquinolones; tetracyclines; acridine dye; phenicols	antibiotic efflux	resistance‐nodulation‐cell division (RND) antibiotic efflux pump
		86.24	1900	1638.6	*MexK*	macrolides; tetracyclines; triclosan	antibiotic efflux	resistance‐nodulation‐cell division (RND) antibiotic efflux pump
		90.36	500	451.8	*MexT*	fluoroquinolones; diaminopyrimidines; phenicols	antibiotic efflux	resistance‐nodulation‐cell division (RND) antibiotic efflux pump
		90.97	1200	1091.6	*arnA*	peptides	antibiotic target alteration	pmr phosphoethanolamine transferase
		91.13	700	637.9	*Escherichia coli EF‐Tu mutants conferring resistance to Pulvomycin*	elfamycins	antibiotic target alteration	elfamycin resistant EF‐Tu
		91.13	700	637.9	*Escherichia coli EF‐Tu mutants conferring resistance to Pulvomycin*	elfamycins	antibiotic target alteration	elfamycin resistant EF‐Tu
		91.88	400	367.5	*Pseudomonas aeruginosa CpxR*	macrolides; fluoroquinolones; monobactam; aminoglycosides; carbapenem	antibiotic efflux	resistance‐nodulation‐cell division (RND) antibiotic efflux pump
	Strict	100.55	200	201.1	*Pseudomonas aeruginosa soxR*	fluoroquinolones; cephalosporins; glycylcycline; penams; tetracyclines	antibiotic target alteration; antibiotic efflux	ATP‐binding cassette (ABC) antibiotic efflux pump; major facilitator superfamily (MFS) antibiotic efflux pump; resistance‐nodulation‐cell division (RND) antibiotic efflux pump
		101.17	750	758.8	*adeF*	fluoroquinolones; tetracyclines	antibiotic efflux	resistance‐nodulation‐cell division (RND) antibiotic efflux pump
		101.5	200	203	*FosA*	fosfomycin	antibiotic inactivation	fosfomycin thiol transferase
		105.8	600	634.8	*Acinetobacter baumannii AbaQ*	fluoroquinolones	antibiotic efflux	major facilitator superfamily (MFS) antibiotic efflux pump
		107.45	200	214.9	*Neisseria gonorrhoeae folP with mutation conferring resistance to sulfonamides*	sulfonamides	antibiotic target alteration	sulfonamide resistant dihydropteroate synthase folP
		178.99	750	1342.4	*adeF*	fluoroquinolones; tetracyclines	antibiotic efflux	resistance‐nodulation‐cell division (RND) antibiotic efflux pump
N71	Loose	80.39	1950	1567.7	*MexB*	macrolides; fluoroquinolones; monobactam; carbapenem; cephalosporins	antibiotic efflux	resistance‐nodulation‐cell division (RND) antibiotic efflux pump
		80.59	1900	1531.2	*TriC*	triclosan	antibiotic efflux	resistance‐nodulation‐cell division (RND) antibiotic efflux pump
		81.96	1900	1557.3	*MexW*	macrolides; fluoroquinolones; tetracyclines; acridine dye; phenicols	antibiotic efflux	resistance‐nodulation‐cell division (RND) antibiotic efflux pump
		84.4	800	675.2	*OprN*	fluoroquinolones; diaminopyrimidines; phenicols	antibiotic efflux	resistance‐nodulation‐cell division (RND) antibiotic efflux pump
		87.07	1900	1654.4	*MexK*	macrolides; tetracyclines; triclosan	antibiotic efflux	resistance‐nodulation‐cell division (RND) antibiotic efflux pump
		90.36	700	632.5	*Escherichia coli EF‐Tu mutants conferring resistance to Pulvomycin*	elfamycins	antibiotic target alteration	elfamycin resistant EF‐Tu
		90.55	1200	1086.6	*arnA*	peptides	antibiotic target alteration	pmr phosphoethanolamine transferase
		90.68	500	453.4	*MexT*	fluoroquinolones; diaminopyrimidines; phenicols	antibiotic efflux	resistance‐nodulation‐cell division (RND) antibiotic efflux pump
		92.45	400	369.8	*Pseudomonas aeruginosa CpxR*	macrolides; fluoroquinolones; monobactam; aminoglycosides; carbapenem	antibiotic efflux	resistance‐nodulation‐cell division (RND) antibiotic efflux pump
		92.85	200	185.7	*FosA*	fosfomycin	antibiotic inactivation	fosfomycin thiol transferase
	Strict	102.51	750	768.8	*adeF*	fluoroquinolones; tetracyclines	antibiotic efflux	resistance‐nodulation‐cell division (RND) antibiotic efflux pump
		105.28	600	631.7	*Acinetobacter baumannii AbaQ*	fluoroquinolones	antibiotic efflux	major facilitator superfamily (MFS) antibiotic efflux pump
		106.3	200	212.6	*Pseudomonas aeruginosa soxR*	fluoroquinolones; cephalosporins; glycylcycline; penams; tetracyclines	antibiotic target alteration; antibiotic efflux	ATP‐binding cassette (ABC) antibiotic efflux pump; major facilitator superfamily (MFS) antibiotic efflux pump; resistance‐nodulation‐cell division (RND) antibiotic efflux pump
		111.1	200	222.2	*Neisseria gonorrhoeae folP with mutation conferring resistance to sulfonamides*	sulfonamides	antibiotic target alteration	sulfonamide resistant dihydropteroate synthase folP
		179.65	750	1347.4	*adeF*	fluoroquinolones; tetracyclines	antibiotic efflux	resistance‐nodulation‐cell division (RND) antibiotic efflux pump

Abbreviation: ARO, Antibiotic Resistance Ontology.

^a^ Some results involved more than 5 different drug classes for some of the matches. In this table, only the first 5 classes are shown in those cases.

The most frequent matches were for genes against fluoroquinolones and the antibacterial agent triclosan, followed by resistance genes for peptide antibiotics, macrolides and diaminopyrimidines. The number of hits varied between isolates but was similar for isolates of the same genus. The *Micromonospora* isolates (S3 and N18) had five hits each, while the *Pseudomonas* isolates (N40 and N71) had 17 and 15 hits, respectively, and the *Pedobacter* isolate N36a had 4 hits. Strict matches (with percentage scores ≥100%) were found only for isolates N18 (1/5 hits), N40 (6/17 hits), and N71 (6/15 hits) (Table 4).

In general, CARD identified the most frequent resistances were genes against fluoroquinolones, tetracyclines, macrolides and penams. In addition, the most common mechanism of resistance was antibiotic efflux (30/46 refined matches), followed by antibiotic target alteration (16/46), target protection (2/46) and antibiotic inactivation (2/46) (Table 4). These findings were supported by the Bakta analyses.

The *Micromonospora* spp. had matches for resistance mostly against macrolides and penams, which explain the NWT phenotypes for penicillin and erythromycin. Further, Bakta analysis identified genes involved in aminoglycoside and fosfomycin resistance. In addition, the *vanZ* and *vanW* genes, belonging to a glycopeptide‐resistance cluster, was found in both isolates. For none of these resistance predictions, a NWT phenotype could be associated. The most common resistant mechanisms were alteration of the antibiotic's target followed by antibiotic efflux, for example, the AcrA/B multidrug efflux pump. The *Pedobacter* isolate had loose matches for resistance against fluoroquinolones, which was also phenotypically confirmed; while the WT for tetracyclines was opposing with the genomic resistance prediction. Not all NWT phenotypes could be directly associated with specific resistance genes, but a general antibiotic efflux could be predicted through carriage of the AcrA/B multidrug efflux pump genes. Further, the *Pseudomonas* spp. showed matches for resistance against fluoroquinolones, tetracyclines, macrolides, phenicols and fosfomycin. Likewise as for the *Pedobacter* isolate, various general resistance mechanisms, genomically identified, as antibiotic efflux, antibiotic target alteration and antibiotic inactivation can explain the observed NWT phenotypes. Finally, matches for resistance against elfamycin antibiotics was found for isolates S3, N18 and N36a as well as sulphonamide antibiotics for S3, N18, N40 and N71. However, no antibiotic from these drug classes were tested and subsequently no corresponding phenotype could be observed.

### Identification of other resistance traits

Additional to the antibiotic resistance prediction, Bakta was used to screen for genes involved in resistances against metals and disinfectant compounds. In all isolates, the workflow could identify genes involved in resistance to metals. The *Micromonospora* spp. isolates showed genes involved in copper (*copC/D*), tellurite (*terB/C*) and cadmium (*cadD*), as well as multiple metal cation (Fe/Co/Zn/Cd) efflux pump (*fieF*). The *Pedobacter* isolate additionally carried a mercury resistance gene (*merC*) as well as a copper and silver efflux pump (*cusA*). The two *Pseudomonas* spp. isolates showed the highest amount of genes involved in metal resistances against zinc, silver, tellurite (*terB/C*), manganese, arsenic (*arsH*), copper (*copC/D*), nickel and cobalt (*cnrA*) and cobalt‐zinc‐cadmium (*czcA*). Further, both *Micromonospor*a isolates as well as the *P. aeruginosa* isolate N71 carried *ermB*/*qacA* genes that are involved in resistance against quaternary ammonium compounds.

## DISCUSSION

Mechanisms of antibiotic resistance new to science are constantly emerging from environmental reservoirs and threatening to render our diminished arsenal of last‐resort antibiotics ineffective (Surette & Wright, 2017; Wright, 2010). This is increasingly likely as global warming destabilizes ecosystems and globalization increases access to areas that were previously remote and pristine (Butler, 2012; Lindgren et al., 2012). Our study intended to investigate the antimicrobial resistance capabilities of putatively virulent bacteria isolated in Arctic soil. These isolates, including Gram‐positives and Gram‐negatives, were selected due to their ability of haemolysis at 37°C. To get insights into the resistance traits of these isolates, phenotypic assays were realized, as well as in silico gene predictions based on whole‐genome sequencing.

Environmental microbiology is lacking specific data and breakpoints for antimicrobial testing and interpretation, so we tried to make use of clinical guidelines (Larsson & Flach, 2022). The testing was aligned to EUCAST guidelines, and we used ECOFF values to classify the isolates as wild‐type (WT) when the isolate was phenotypically susceptible to a specified antimicrobial agent or non‐WT (NWT) when there was phenotypical resistance to a specified antimicrobial agent, according to EUCAST guidelines.

Genome data‐based investigations revealed that the most frequent drug classes against which resistance determinants were found were fluoroquinolones, tetracyclines, macrolides and penams (penicillins), which was consistent with the phenotypic results. It should be noted, that the gene prediction for environmental bacteria was not expected to show comparable good results as, for example, for healthcare‐associated species. This fact is reasoned by the focus of the underlying databases, mostly missing data for the entire One Health and environmental microbiology sphere. This was one main rationale for this study to use different annotation and prediction databases and try to consolidate the obtained results, also with phenotypical MIC testing. By this we were not able to identify potential new resistance genes or mechanisms that were validly associated with MIC results.

Analysis with CARD showed that isolates S3 and N18 had the most resistance against macrolides and penams, which was confirmed by their classification into the NWT for both ERY and PEN. In this study, three of the isolates (S3, N18 and N36a) belonged to the NWT for ERY. Both S3 and N18 had loose hits with genes *poxtA* and *mtrA*, which confer multidrug resistance, including oxazolidinone antibiotics (Crowe‐McAuliffe et al., 2022). In *Micromonospora* spp. isolates, the analysis further identified genes conferring resistance to macrolide antibiotics mediated by the ABC‐F ATP‐binding cassette, that unlike other ABC cassettes, confers resistance but by protecting the ribosome, not by efflux (Sharkey et al., 2016).

Despite both *Micromonospora* spp. having the same determinants for resistance against tetracycline, only S3 was classified as NWT, possibly due to a new mechanism or differential gene expression compared to N18. Additionally, EUCAST establishes a 0.5 mg L^−1^ breakpoint for non‐species‐related susceptibility to the aminoglycoside GEN. Our experiments show that the only isolate with <0.5 mg L^−1^ is S3, deeming all others resistant. Moreover, N18 belonged to the WT for CIP, despite being considered clinically resistant according to EUCAST with an MIC value >0.5 mg L^−1^.

Regarding the last‐resort treatment option colistin for *Pseudomonas* spp., the isolate N40 showed a low MIC while N71 a high MIC. Interestingly both isolates carried the *arnA* gene, which confers resistance to polymyxin antibiotics and colistin (Gatzeva‐Topalova et al., 2005; Lee et al., 2016). Polymyxin‐resistance is well known in *Pseudomonas* species and has quite diverse genetic origins; therefore, our findings are eligible taking in consideration that isolate N40 might be a new species (Jeannot et al., 2021).

The *Pedobacter* sp. N36a was in 80% NWT for antibiotics tested. CARD showed that N36a carries resistance genes against fluoroquinolones and tetracyclines, despite showing several resistances to a variety of the antimicrobials used in the study; highlighting the importance of combined in silico and in vitro investigations. The *Pedobacter* N36a was classified as multidrug resistant and showed resistance to β‐lactams, colistin, aminoglycosides and ciprofloxacin. Our results show the relevance of *Pedobacter* strains, as these are regarded in literature as “environmental superbugs” to highlight the reservoirs of resistance determinants in the Arctic biosphere (Viana et al., 2018). These resistances might be mediated by efflux pumps, as sequencing showed, a result that could not be inferred from phenotypic assays alone (Leclercq et al., 2013). We further observed that N36a could belong to a novel *Pedobacter* species, bearing the potential of relevance as upcoming opportunistic pathogen.

Further, the genomic datasets were used to screen for other types of resistance traits, as against metals and disinfection compounds. The combined analyses by CARD and PATRIC identified matches for resistance against triclosan, a synthetic antimicrobial agent widely used in the cosmetic and disinfection industries, responsible for membrane leakage and transcription alteration in prokaryotic cells (Carey & McNamara, 2014; Yim et al., 2006). Additional, Bakta analyses revealed genes involved in resistance to quaternary ammonium compounds (QACs), which are important disinfectants. This is interesting, since some of the mechanisms conferring resistance to triclosan are responsible for resistance to several classes of antibiotics, for example, tetracycline (Boyce, 2023; Carey & McNamara, 2014; Varela et al., 2023). The presence of these genes is quite common for several nosocomial and environmental genera, including *P. aeruginosa* (Wassenaar et al., 2015). Recent studies highlight the urge to co‐investigate metal tolerance/resistance genes in environmental bacteria, especially from remote and less‐studied regions. So, identifying metal and heavy‐metal resistance genes in our isolates was not unexpected, for example, *Pseudomonas* species are well known for their copper tolerance (Behrendt et al., 2007; Sajjad et al., 2023). However, over the last years this became an important topic for antimicrobial resistance (Vats et al., 2022). Studies identified mechanisms of co‐selection, since heavy‐metals pose a comparable selection pressure for bacterial physiology (Liu et al., 2022; Schürmann et al., 2024). Some of these resistances, as tellurite, seem to show a correlation to pathogenicity (Turkovicova et al., 2016). All (heavy‐) metal resistance genes are known to enhance the general stress physiology and resilience of bacteria, enabling them to survive and thrive also with hygiene measures in man‐built environments, including hospitals (Bombaywala et al., 2021; Virieux‐Petit et al., 2022).

With this study we were able to characterize the resistome of five haemolytic strains isolated from Arctic soils. Determining MICs for non‐clinical isolates is of importance, as few studies have been focussing in this topic, resulting in a lack of data and uncertainties in interpretation (Larsson & Flach, 2022). The combined in vitro data with genomic resistance prediction was an added value so for, but also revealed that missing databases and publications can lead to difficult to interpret results, for example, reasonably ECOFF approximations or several loose hits in gene prediction (Edwards et al., 2020). Nevertheless, the study results represent an impetus to further study the surprisingly complex Arctic soil microbiome, which could increasingly move into focus due to progressions in both, the climate change and the global interlinking (Makowska‐Zawierucha et al., 2024). Makowska‐Zawierucha et al. highlighted resistance spread of resistance traits in the One Health context, especially focusing on plasmids. Fortunately, in our study isolates no plasmids were detected, so this threat of gene spread and exchange is minor for our specific setting. But as we stated in the introduction, due to human movements and actions, as well as migration of animals, as birds, Arctic soil bacteria are able to migrate to new environments, on a global scale eventually (Cowan et al., 2011; Gattinger et al., 2024; Makowska‐Zawierucha et al., 2024). In total, our study poses one piece in a jigsaw of understanding the environmental occurrence of resistances and ultimately to a better understanding of the Arctic resistome.

## CONCLUSIONS

As Arctic soils experience both profound warming and increased pressures from human activities, the scope for transfer of antimicrobial resistant bacteria is increased. The resistance determinants reported in this study could therefore pose a threat if the responsible genes are transferrable between species or become widespread. Observed resistance phenotypes for which we could not find genetic mechanisms in current databases represent potential threats. Moreover, the fact that these soil bacteria were able to grow over 30°C and produce haemolytic enzymes, enhancing fitness and additionally pose the possibility that these Arctic bacteria become opportunistic pathogens for human beings. The results presented here contribute to the understanding of virulence‐associated phenotypes of Arctic bacteria and their resistance assets. Further we provide valuable information for assessing possible threats for other environments also heavily affected by climate change.

## AUTHOR CONTRIBUTIONS

**Diana C. Mogrovejo‐Arias:** Conceptualization; investigation; writing – original draft; writing – review and editing; formal analysis; visualization. **Melanie C. Hay:** Investigation; writing – review and editing; formal analysis. **Arwyn Edwards:** Resources; writing – review and editing; supervision. **Andrew C. Mitchell:** Resources; supervision; writing – review and editing. **Jörg Steinmann:** Writing – review and editing; supervision; validation. **Florian H. H. Brill:** Supervision; resources; writing – review and editing. **Bernd Neumann:** Writing – original draft; writing – review and editing; investigation; formal analysis; data curation; software.

## CONFLICT OF INTEREST STATEMENT

Florian H. H. Brill is the managing director of Dr. Brill + Partner GmbH. Diana C. Mogrovejo‐Arias is employed by Dr. Brill + Partner GmbH and declares no conflict of interest. All other authors declare no conflicts of interest.

## Supporting information


**Table S1.** Whole genome assembly metrics for the 5 isolates sequenced in this study. Quality data was calculated using QUAST and closest related species were obtained using the GTDB‐tk tool on Kbase. ANI, Average Nucleotide Identity.
**Table S2.** Genome annotation, protein features and specialty genes obtained with the Comprehensive Genome Analysis service of PATRIC. For the specialty genes, homology to known transporters, virulence factors, drug targets, and antibiotic resistance genes is shown with the number of genes and the specific source database where the homology was found.

## Data Availability

The data that support the findings of this study are openly available in OmicsDI at https://www.omicsdi.org/dataset/omics_ena_project/PRJNA659552. This study uses genomic data first published by Mogrovejo‐Arias et al. (2020) (DOI: https://doi.org/10.1007/s12665-020-8853-4) in which all accession data is provided. In addition, genome sequences generated from the present study have been deposited in the National Center for Biotechnology Information (NCBI) database under the Bioproject number PRJNA659552. Additional data is available from the authors upon request.
