# Supplementary material for: Investigating the resistome of haemolytic bacteria in Arctic soils
Source: Environ Microbiol Rep. 2024 Oct 23;16(5):e70028. doi: 10.1111/1758-2229.70028 (PMC11497493; doi:10.1111/1758-2229.70028)
Supplement: Supplementary file 1 — Table S1. Whole genome assembly metrics for the 5 isolates sequenced in this study. Quality data was calculated using QUAST and closest related species were obtained using the GTDB‐tk tool on Kbase. ANI, Average Nucleotide Identity. Table S2. Genome annotation, protein features and specialty genes obtained with the Comprehensive Genome Analysis service of PATRIC. For the specialty genes, homology to known transporters, virulence factors, drug targets, and antibiotic resistance genes is shown with the number of genes and the specific source database where the homology was found. [file EMI4-16-e70028-s001.docx]

**Table S1.** Whole genome assembly metrics for the 5 isolates sequenced in this study. Quality data was calculated using QUAST and closest related species were obtained using the GTDB-tk tool on Kbase. ANI: Average Nucleotide Identity

| **Sample ID** | **NCBI BioSample number** | **NCBI Genome Accession number** | **Contigs (**≥**0bp)** | **Contigs (**≥**1000bp)** | **Largest contig (bp)** | **Total length**  **(bp)** | **GC (%)** | **N50** | **N75** | **L50** | **L75** | **Closest related species** | **% ANI** |
| --- | --- | --- | --- | --- | --- | --- | --- | --- | --- | --- | --- | --- | --- |
| S3 | SAMN15915235 | [JACSZY000000000](https://www.ncbi.nlm.nih.gov/nuccore/JACSZY000000000) | 696 | 634 | 116704 | 7262638 | 72.72 | 18139 | 10401 | 126 | 260 | *Micromonospora aurantiaca* | 98.61 |
| N18 | SAMN15915236 | [JACSZX000000000](https://www.ncbi.nlm.nih.gov/nuccore/JACSZX000000000) | 539 | 478 | 116475 | 6962593 | 72.74 | 24232 | 13053 | 90 | 187 | *Micromonospora chalcea* | 98.72 |
| N36a | SAMN15915262 | [JACSZW000000000](https://www.ncbi.nlm.nih.gov/nuccore/JACSZW000000000) | 86 | 39 | 834625 | 5235388 | 40.5 | 465834 | 223025 | 5 | 8 | *Pedobacter sp.* | 90.34 |
| N40 | SAMN15915370 | [JACSZV000000000](https://www.ncbi.nlm.nih.gov/nuccore/JACSZV000000000) | 109 | 93 | 313368 | 6206379 | 58.88 | 112392 | 67070 | 19 | 37 | *Pseudomonas sp.* | 91.24 |
| N71 | SAMN15915371 | [JACSZU000000000](https://www.ncbi.nlm.nih.gov/nuccore/JACSZU000000000) | 96 | 64 | 479071 | 6157248 | 60.81 | 202875 | 115070 | 10 | 20 | *Pseudomonas lurida* | 99.26 |

**Table S2.** Genome annotation, protein features and specialty genes obtained with the Comprehensive Genome Analysis service of PATRIC. For the specialty genes, homology to known transporters, virulence factors, drug targets, and antibiotic resistance genes is shown with the number of genes and the specific source database where the homology was found

| **Isolate** | **CDS** | **Hypothetical proteins** | **Proteins with functional assignments** | **Proteins with EC number assignments** | **Proteins with GO assignments** | **Specialty genes** | | | |
| --- | --- | --- | --- | --- | --- | --- | --- | --- | --- |
|  |  |  |  |  |  | **Antibiotic resistance** | **Transporter (TCDB)** | **Virulence factor** | **Drug target** |
| S3 | 7226 | 2882 | 4344 | 1311 | 1113 | C: 2  P: 42 | 5 | VC: 2  PV: 3 | D: 3 |
| N18 | 6768 | 2568 | 4200 | 1286 | 1088 | C: 2  P: 43 | 3 | VC: 2  PV: 2 | D: 3 |
| N36a | 4735 | 1873 | 2862 | 935 | 809 | P: 28 | 1 | VC: 1  PV: 1 | 0 |
| N40 | 5745 | 1280 | 4465 | 1219 | 1046 | C: 4  P: 70 | 76 | VF: 25  VC: 27 | D: 27  T: 6 |
| N71 | 5690 | 1120 | 4570 | 1294 | 1112 | C: 4  P: 72 | 72 | VF: 27  VC: 23  PV: 2 | D: 26  T: 6 |
| CDS: protein coding sequences; EC: Enzyme Commission; GO: Gene Ontology; C: CARD; P: PATRIC; TCDB: transporter classification database; VF: virulence factor database; VC: Victors; PV: PATRIC_VF; D: DrugBank; T: therapeutic target database | | | | | | | | | |
